# Cortical circuit dynamics underlying motor skill learning: from rodents to humans

**DOI:** 10.3389/fnmol.2023.1292685

**Published:** 2023-10-26

**Authors:** Emily Kogan, Ju Lu, Yi Zuo

**Affiliations:** Department of Molecular, Cell and Developmental Biology, University of California, Santa Cruz, Santa Cruz, CA, United States

**Keywords:** motor learning, cross-species, primary motor cortex, neuron, inhibitory interneuron, synapse, dendritic spine

## Abstract

Motor learning is crucial for the survival of many animals. Acquiring a new motor skill involves complex alterations in both local neural circuits in many brain regions and long-range connections between them. Such changes can be observed anatomically and functionally. The primary motor cortex (M1) integrates information from diverse brain regions and plays a pivotal role in the acquisition and refinement of new motor skills. In this review, we discuss how motor learning affects the M1 at synaptic, cellular, and circuit levels. Wherever applicable, we attempt to relate and compare findings in humans, non-human primates, and rodents. Understanding the underlying principles shared by different species will deepen our understanding of the neurobiological and computational basis of motor learning.

## 1. Introduction

Muscle-based motor systems are evolutionarily very ancient, present in all metazoans except sponges (Seipel and Schmid, 2005; Wang et al., 2022). In an ever-changing, unpredictable environment, it is advantageous to have a variety of motor functions and to be able to generate motor outputs adaptively. Mammals arguably possess the richest motor repertoire, and have the capability to change or improve motor performances through practice, a process generally referred to as motor learning (Shmuelof and Krakauer, 2011). Mammalian motor behaviors engage a complex network of brain regions, including the neocortex, the basal ganglia, the motor thalamus, the cerebellum, and brainstem and midbrain areas such as the ventral tegmental area (VTA), the substantia nigra pars compacta (SNc), the periaqueductal gray area (PAG), the vestibular nuclei, and the pedunculopontine nucleus (PPN) (Suryanarayana et al., 2022). Particularly, placental mammals possess at least one separate cortical motor area (Krubitzer, 2007). The primary motor cortex (M1) serves as a control hub of this network, sending commands that enable the flexible recruitment of lower motor neurons in the spinal cord, which underlies the ability to learn and to perform novel tasks (Shmuelof and Krakauer, 2011). Its functions are subserved by the local circuit as well as the long-range connections with other brain regions. M1 has a laminar structure, containing many of the same neuronal elements of the canonical microcircuit as in other neocortical areas (Douglas and Martin, 2004). Traditionally, it is believed that M1 is cytoarchitectonically distinct by the lack of layer (L) 4 (Geyer et al., 2000), which mainly consists of granular cells and is the principal target of thalamic inputs in sensory cortices. Some recent studies (Garcia-Cabezas and Barbas, 2014; Yamawaki et al., 2014; Bopp et al., 2017), however, suggest the existence of a *bona fide* L4 in M1 of mice and rhesus monkeys, which echoes Ramón y Cajal’s description of a L4 in the human motor cortex (Ramón y Cajal, 1899).

M1 receives inputs from several thalamic nuclei (thalamocortical or TC inputs) and other cortical areas (corticocortical or CC inputs). TC inputs exhibit a sub-region- and layer-specific distribution. The ventral medial nucleus (VM) and the anterior medial nucleus (AM) of the thalamus project to L1, the ventral anterior/ventral lateral nucleus (VA/VL) projects to L1 and L2/3, and the posterior nucleus (PO) innervates L2/3 and L5 (Hooks et al., 2013; Kuramoto et al., 2015; Tanaka et al., 2018; Hasegawa et al., 2020; Munoz-Castaneda et al., 2021). CC inputs also exhibit sub-region- and layer-specificity. For example, L5 neurons in the mouse rostral forelimb area (RFA) of the motor cortex receive inputs from L2/3 and L5A, but not L5B, neurons in the caudal forelimb area (CFA); conversely, CFA L5 neurons receive inputs from RFA L5B neurons (Hira et al., 2013). In the rat brain, it has been reported (Ueta et al., 2014) that projections from the secondary motor cortex (M2) to M1 mostly arise from pyramidal neurons (PyrNs) in lower L2/3 and L5A. Among them, M2 L5A neurons predominantly send their axons to L1 of M1, whereas M2 L2/3 neurons also innervate L2/3 of M1. Furthermore, in the mouse vibrissal M1 (vM1), axons from the vibrissal primary somatosensory cortex (vS1) preferentially innervate L2/3 and L5A neurons, providing only weak inputs to L5B and L6 neurons (Mao et al., 2011). A later study by the same group (Hooks et al., 2015) showed with dual-channel optogenetic stimulation that individual L2/3 neurons in the mouse vM1 receive inputs from both vS1 and the posteromedial nucleus of the thalamus (POm). Taken together, such anatomical specificity suggests that neurons in different layers of M1 integrate different information streams for motor functions.

**Figure 1:**
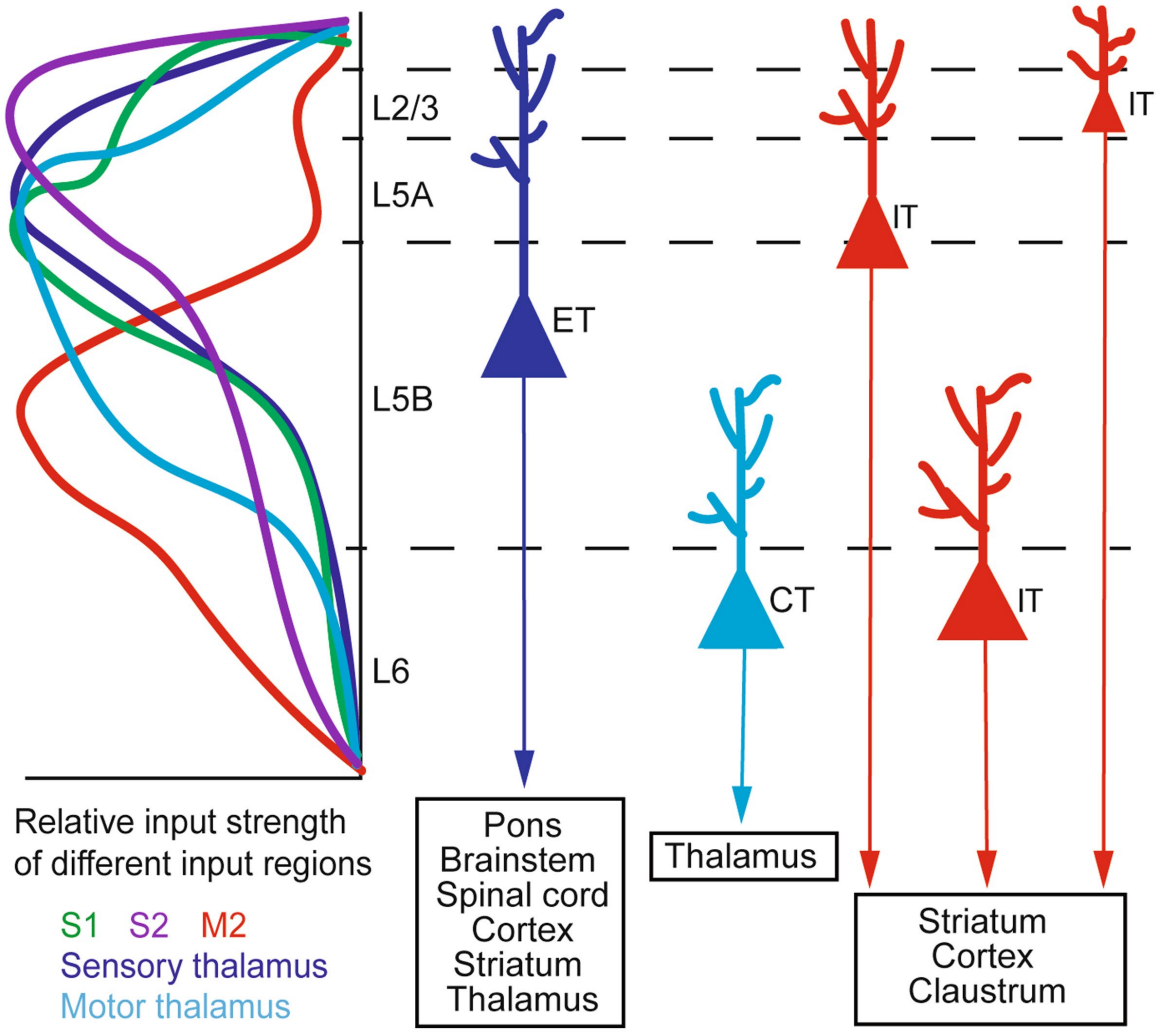
A schematic of M1 input–output organization. Left: the laminar distribution of inputs from major sources to M1, shown as relative input strength. Right: targets of major types of M1 output neurons. ET, extratelencephalic neurons (traditionally termed pyramidal tract or PT neurons); IT, intratelencephalic neurons; CT, corticothalamic neurons. Adapted from Baker et al. (2018, licensed under CC-BY 4.0), Hooks et al. (2013, licensed under CC-BY-NC-SA 3.0), and BRAIN Initiative Cell Census Network (BICCN) (2021, licensed under CC-BY 4.0).

M1 sends outputs to many brain regions. Among the main projection neurons in M1, pyramidal tract (PT) neurons send axons down the white matter tract in the brainstem and innervate a variety of regions within and outside the telencephalon, including the spinal cord, pons, striatum, brainstem, and thalamus; intratelencephalic (IT) neurons, in contrast, project exclusively within the telencephalon to the cortex and striatum (Baker et al., 2018). A special sub-class of PT neurons are the corticomotoneuronal (CM) cells, which directly synapse onto spinal cord alpha motor neurons controlling hand and finger muscles. They are unique to dexterous primates, which they work synergistically with evolutionarily more ancient descending pathways to generate highly dexterous movements (Lemon, 2008; Yoshida and Isa, 2018; Strick et al., 2021). In rodents CM connections are present only transiently and are eliminated shortly after birth (Gu et al., 2017; Murabe et al., 2018). Besides projecting to different targets, one recent study also suggests that corticostriatal projections from M1 IT neurons exhibit a higher degree of topographic stereotypy than those from PT neurons (Hooks et al., 2018). A schematic of the organization of major M1 inputs and outputs is given in Figure 1. For a more comprehensive description of M1 input and output patterns, see (Hooks et al., 2013; Baker et al., 2018; BRAIN Initiative Cell Census Network (BICCN), 2021; Munoz-Castaneda et al., 2021); for details of cortical local circuitry, see (Harris and Shepherd, 2015).

## 2. M1 is necessary for motor learning and dexterity but not for simple movements

Previous studies across species have demonstrated that M1 plays an essential role in motor learning. An early human study (Schlaug et al., 1994) used positron emission tomography (PET), which uses radioactive tracers to measure physiological processes such as metabolism and blood flow (Raichle, 1983), to identify cerebral structures activated in a task in which participants carried out a complex sequence of finger tapping. It showed that the contralateral sensorimotor area had the highest activation, with other cortical and subcortical areas variably implicated. Another study trained human subjects to move fingers in specific orders paired with specific rhythms and found through functional magnetic resonance imaging (fMRI) that contralateral M1 encodes the learned finger movement sequences and rhythms as an integrated unit (Kornysheva and Diedrichsen, 2014). Later, it was discovered that bilateral transcranial direct current stimulation (tDCS) to the motor cortex substantially improved the human subject’s learning in a unimanual sequence movement task compared to unilateral or sham stimulation (Waters et al., 2017). Upregulation of M1 excitability by transcranial magnetic stimulation (TMS) (Hallett, 2000) also improved learning in a non-dominant hand digit sequence task (Narayana et al., 2014) and during a serial reaction time task (SRTT, commonly used to investigate motor learning in humans) (Nitsche et al., 2003). On the other hand, inactivating M1 interferes with motor learning. For example, Muellbacher et al. trained humans to perform fast finger movement and measured movement acceleration and muscle force generation. They found that low-frequency, repetitive TMS to M1 acutely disrupted the retention of learned behavior, without affecting basal motor behaviors or subsequent motor learning, suggesting that M1 is specifically engaged in the early phase of motor skill consolidation (Muellbacher et al., 2002). Another study found that inhibitory TMS through continuous theta burst stimulation to M1 impaired motor learning in SRTT. This effect is believed to result from a decreased functional connectivity between M1 and other brain regions relevant to the task (Steel et al., 2016).

In rodent models, researchers have tried to identify brain regions active during motor learning by immunostaining Fos, the protein product of the immediate-early gene cFOS, as a proxy for neuronal activation. An early study (Kleim et al., 1996) found that Fos expression was significantly higher in M1 L2/3 of rats trained on an acrobatic task (traversing a series of 10 obstacles including a rotating cylinder, a suspended chain, wooden blocks, dowel rods of varying diameters, etc.), which requires a wide variety of fine motor skills, compared to control animals. Furthermore, Fos signal was greater in brains collected during skill acquisition than in those collected during task performance, suggesting that M1 is the most active during the early phase of learning, consistent with findings in human experiments (Muellbacher et al., 2002). Notably, training on complex motor behaviors (running on a wheel with an irregular rung pattern) activates M1 stronger than simple motor training (running on an accelerating rotating rod or “rotarod”), as shown by Fos immunostaining (Nagai et al., 2017).

Interestingly, while accumulating evidence suggests that M1 is crucial for motor learning and dexterity, it seems more or less dispensable for simpler, “innate” movements. Early studies have shown that lesioning the motor cortex (Castro, 1972; Whishaw, 2000) impairs dexterous movements in rats. Unilateral partial or complete pyramidal sections, which disrupt the corticospinal tract (CST, the major neural pathway connecting M1 to the spinal cord), likewise impair skilled reaching in rats (Whishaw et al., 1993; Piecharka et al., 2005). Similarly, interrupting the CST in monkeys by bilateral lesioning at the upper medullary level impairs dexterity (Lawrence and Kuypers, 1968). However, simpler movements such as running in a maze or cage are generally not impaired by cortical lesions encompassing M1 (Franz and Lashley, 1917; Maier, 1935). A study on rhesus monkeys also showed that after complete removal of sensorimotor areas including M1, walking and jumping recovered over time but dexterity did not (Passingham et al., 1983). To avoid the confounding effect of M1 lesion on dexterity during motor learning, Kawai et al. created a motor sequence task, in which the rat learns to press a lever twice in a prescribed temporal sequence to obtain a water reward (Kawai et al., 2015). As dexterity is not explicitly required, this task dissociates the learning and execution of motor sequences from the generation of dexterous movements. Strikingly, although pre-training M1 lesions prevented rats from acquiring the motor sequence, post-training M1 lesions did not affect the execution of the acquired motor sequence. Consistent with this, M1 inactivation through optogenetic activation of inhibitory interneurons impairs the mouse’s motor performance in early and mid, but not late, stages of learning a joystick-press task (Hwang et al., 2019). Moreover, a recent study (Serradj et al., 2023) showed that learning a “precise” motor task (isometric pull with a specific force) remodeled the activity pattern of relevant M1 corticospinal neurons in mice, whereas learning an “imprecise” task (pull without the need for precise force adjustment) did not. Bilateral pyramidotomy or optogenetic silencing of corticospinal neurons disrupted the performance in the precise task but largely spared the imprecise task. Taken together, these studies highlight M1’s role in learning new, complex motor skills rather than performing routine motor tasks.

## 3. Motor learning induces gross anatomical changes in M1

Both human and animal studies have revealed that motor learning induces macroscopic anatomical changes in M1. Several works have compared the brains of musicians (professional and amateur keyboard players) and non-musicians, as playing a musical instrument requires one to learn complex fine movement patterns and to rehearse them over time. Using voxel-based morphometry (Ashburner and Friston, 2000), an automated technique to identify brain anatomy differences by statistical analysis of high-resolution structural MRI data, Gaser and Schlaug found a significant positive correlation between musician status and the volume of gray matter in several brain regions including M1, with professional musicians having the largest volume (Gaser and Schlaug, 2003). Analogously, world-class gymnasts have a larger volume of gray matter in the precentral gyrus (M1) than non-gymnast controls, and among gymnasts, larger gray matter volume correlates with higher average scores in routines they competed (Fukuo et al., 2020). In concert pianists, diffusion tensor imaging (DTI) discovered a significant correlation between the practice time and the fractional anisotropy (FA) (Bengtsson et al., 2005). FA is a widely-used measure that reflects axonal fiber density, diameter, and myelination in the white matter (Le Bihan, 2003). Interestingly, practice time during different age periods–childhood, adolescence, or adulthood–correlates with FA in different sets of brain regions. In particular, childhood practice time correlates with FA in the posterior limbs of the internal capsule, which contain CST fibers that are critically important for independent finger movements in humans and other primates (Bengtsson et al., 2005). Furthermore, learning a new motor skill can modify the motor cortex on a shorter time scale. Human subjects practicing left hand finger-thumb opposition movement for 4 weeks showed an increase in cortical thickness in the right post-central gyrus, consistent with the motor representation of trained fingers (Sale et al., 2017). Diffusion MRI tractography has further revealed a significantly increased FA in the right CST, with no change in the left CST, in subjects undergoing such training (Reid et al., 2017). A recent study comparing DTI results with myelin water imaging (MWI) suggests that increased myelination plays a central role in motor learning-induced white matter plasticity (Kirby et al., 2022). Similar macroscopic reorganizations in response to motor learning have also been observed in the rodent M1. An early study reports that consistent use of one forelimb in a reaching task increases the thickness of the contralateral M1 in rats (Diaz et al., 1994). The increase in motor cortical thickness has also been observed in rats subjected to motor learning on an obstacle course, but not in those voluntarily running on a wheel or in inactive controls (Anderson et al., 2002). Recently, it was found that learning a dexterous reaching task induces dynamic retraction and formation of myelin sheaths around learning-activated axons in mouse M1 (Bacmeister et al., 2022). Together, these studies suggest that both gray and white matter undergo structural alterations in motor learning.

Interestingly, humans learning a three-ball juggling task for 3 months show a transient gray matter expansion, which decreases over the following 3 months of non-practice (Draganski et al., 2004). Another study on right-handed subjects practicing left-hand writing found that the gray matter volume increased over the first 4 weeks, but then returned to the baseline level either completely or partially (Wenger et al., 2017b). These results have led to the expansion-renormalization model of plastic changes in the human brain accompanying skill acquisition (Wenger et al., 2017a). The model postulates an initial increase of gray matter structure, such as synaptic growth, followed by a selective elimination of “unwanted” surplus structures, which renormalizes the total volume. It resolves a paradox already recognized by Ramón y Cajal: “[How] can the volume of the brain be maintained if there is a multiplication and a new formation of small terminal branches of dendrites and axonal collaterals?” (Ramón y Cajal, 1894). It also broadly agrees with the observation of learning-associated structural dynamics of synapses in rodents *in vivo* (see below).

## 4. Neuronal representation of learned behavior emerges and evolves with learning

Many studies have attempted to determine how motor commands are encoded at the level of individual neurons or neuronal populations. Evidence abounds that single neurons in primate M1 may encode a variety of kinematic parameters related to movement, such as position (Georgopoulos et al., 1984; Paninski et al., 2004), direction of movement (Georgopoulos et al., 1982), amplitude (Messier and Kalaska, 2000), and acceleration (Ashe and Georgopoulos, 1994). Indeed, a large variable set of finger movements may be decoded from the activity patterns of a relatively small number of neurons in monkey M1 (Ben Hamed et al., 2007). However, as the activity of single M1 neurons may correlate with a wide range of movement parameters and change across behaviors, it leads some researchers to speculate that the apparent “tuning” of individual M1 neuron’s activity to specific movement variables is but coincidental; that it would be more natural to view M1 as a dynamical system that generates and controls movements, in which the activity of individual neurons evolves as part of the system-wide dynamics (Scott, 2008; Churchland et al., 2012). As succinctly put in a Perspective article, “the ultimate role of M1 is to generate movement, not to represent it” (Gallego et al., 2017). An emerging idea is that the parameters to generate movements are encoded in the activity pattern of populations of neurons, which may lie on a low-dimensional manifold (Shenoy et al., 2013; Gallego et al., 2017; Vyas et al., 2020). According to this idea, motor learning would involve a gradual shift in the population activity patterns that leads to the optimization of the motor output.

Previous studies have shown that motor learning leads to functional reorganization of M1, *i.e.*, changes in the topography of motor representations. An fMRI study (Karni et al., 1995) on human subjects learning a motor sequence (finger opposition movement) showed that the activated region in M1 grew as learning proceeded, and after weeks of practice, the active area remained large compared to areas activated upon learning a new task. In squirrel monkeys, learning two tasks–small object retrieval, which requires digit movement, and eyebolt-turning, which requires supination and pronation of the hand–differentially alters the topographic map in M1, as determined by intracortical microstimulation (Nudo et al., 1996). This is corroborated by the finding that skilled reaching (pellet retrieval) elicits an increase in the area of wrist and digit representation in rat M1, whereas unskilled reaching (bar pressing) does not (Kleim et al., 1998). The expansion of distal forelimb representation occurs only after 10 days of training, following significant synaptogenesis (Kleim et al., 2004). On the other hand, as learning progresses, M1 displays the same or even reduced global activation level. A human fMRI study (Wiestler and Diedrichsen, 2013) found that highly distinguishable activation patterns emerged in M1 for trained finger movement sequences without an increase in average activity.

To determine how M1 population activity pattern evolves during motor learning with single neuron resolution, Peters et al. performed *in vivo* two-photon (2P) calcium imaging of L2/3 neurons in the mouse M1 during a lever-press task over 2 weeks (Peters et al., 2014). They found that, during the initial phase of learning, the population of active excitatory neurons expanded and exhibited a variety of activity patterns even when executing similar movements; as learning proceeded, this was refined to a smaller population with more reproducible activity patterns. A later study by the same group (Hwang et al., 2019) confirmed that M1 L2/3 population activity became more consistent across trials from early- to mid-stage of learning a joystick-press task, as kinematic stereotypy rapidly increased. However, over prolonged training (2 months), M1 population activity became again variable despite expert-level performance, which is consistent with the disengagement of M1 from controlling well-established movements. Recently, Hwang et al. further tested this idea by training mice on two tasks requiring distinct sets of muscles (forward vs. downward joystick press) over long term. They found that of the two tasks, the one in which the mouse achieved higher trial-to-trial consistency activated fewer M1 L2/3 neurons, producing weaker and less consistent population activity, and was less affected by optogenetic inactivation of M1 (Hwang et al., 2021). Apparently, M1 relinquishes the control over movements that have achieved perfection through practice.

## 5. Reorganization of M1 excitatory synaptic circuit in motor learning

It is widely accepted that the activity pattern of neurons is fundamentally determined by their synaptic connectivity. A corollary of this idea is that changes in synaptic connections underlie the evolution of neural activity patterns throughout the learning process. Indeed, Ramón y Cajal suggested this possibility as early as in 1894. In the Croonian Lecture delivered before the Royal Society of London, he perspicaciously remarked that, in a use-dependent manner, “associations already established among certain groups of cells would be notably reinforced by means of the multiplication of the small terminal branches of the dendritic appendages and axonal collaterals; but, in addition, completely new intercellular connections could be established thanks to the new formation of [axonal] collaterals and dendrites” (Ramón y Cajal, 1894). Essentially, he envisages that structural plasticity in the synaptic circuit serves as the anatomical substrate of learning.

In the context of motor learning, evidence supporting this idea in M1 emerged almost a century later. In 1996, Greenough et al. used serial electron microscopy (EM) to examine M1 L2/3 neurons in rats trained on acrobatics, and found that their synapse density increased from skill acquisition to the maintenance phase of learning (Kleim et al., 1996). Consistent with this, in rats trained on a pellet reaching task, synapse number per M1 neuron significantly increased at day 7 and day 10 of learning compared to the pre-learning level (Kleim et al., 2004). While such fixed-tissue studies have yielded invaluable information about learning-associated synaptic changes, they cannot follow the dynamic process. The advent of two-photon microscopy (Denk et al., 1990) and transgenic mice with cortical neurons sparsely labeled by fluorescent proteins (Feng et al., 2000) enable researchers to interrogate the structural dynamics of synapse changes in the living brain. Focusing on dendritic spines – tiny protrusions that host the postsynaptic sites of most excitatory synapses in the mammalian cortex – on apical dendrites of L5 PyrNs in sensory cortices, several pioneering works (Grutzendler et al., 2002; Trachtenberg et al., 2002; Zuo et al., 2005; Holtmaat et al., 2006) discovered a rich dynamics of spine formation and elimination under baseline conditions and in response to sensory manipulations. Leveraging the same imaging technique, Xu et al. found a rapid emergence of new spines on M1 L5 PyrNs in mice trained on a pellet reaching task; continued training preferentially stabilized such learning-induced spines, which persisted long after the cessation of training (Xu et al., 2009). A similar phenomenon was reported in mice receiving rotarod training (Yang et al., 2009).

Moreover, spine formation and stabilization are correlated with the acquisition (Xu et al., 2009) and retention (Yang et al., 2009) of learned motor skills. Subsequent studies further demonstrated the positive correlation between spine dynamics and motor learning by molecular or genetic tools. For example, intrathecal application of antibodies against Nogo-A (a membrane protein best known as an inhibitor of axonal outgrowth and regeneration in the central nervous system) increases spine formation and improves learning of single-pellet reaching (Zemmar et al., 2014). Mice over-expressing KCC2 (a neuronal K^+^-Cl^−^ co-transporter) in forebrain excitatory neurons show increased spine formation rate during rotarod training, which is correlated with faster learning and better performance outcome (Nakamura et al., 2019). Similarly, paired immunoglobulin receptor B knockout (PirB^−/−^) mice have increased spine formation rate as well as lower spine elimination rate, and learn skilled reaching faster. Notably, acute blockage of PirB signaling by infusion of a recombinant soluble PirB decoy receptor in wild-type mice recapitulates the spine dynamics phenotype and learning improvement observed in PirB^−/−^ mice, suggesting that the effect is not due to a developmental alteration in the neural circuit (Albarran et al., 2021). To move beyond correlation and establish the causal relationship between the structural potentiation of spines (formation and enlargement) and motor learning, Hayashi-Takagi et al. devised a novel “optoprobe” called AS-PaRac1, which is targeted to potentiated spines and can induce spine shrinkage when activated by light. They expressed AS-PaRac1 in M1 and trained the mice to run on the rotarod. Light activation of AS-PaRac1 after learning caused the potentiated spines to shrink and impaired the animal’s performance on the rotarod. The effect is task-specific, as AS-PaRac1 activation of spines potentiated during beam walking or spontaneously during other movements did not interfere with rotarod performance. Thus, this study provides the first direct evidence that structural changes in M1 synaptic connections are necessary for motor learning (Hayashi-Takagi et al., 2015).

Furthermore, new spines associated with motor learning do not form randomly along the dendrite, but exhibit distinct spatial patterns. On L5 PyrNs, new spines form in clusters in response to motor learning, and the emergence of the second spine in the cluster is associated with the enlargement of the first spine (Fu et al., 2012). Recently, an elegant study (Hedrick et al., 2022) used *in vivo* glutamate imaging to reveal the potentiation of clustered spines exhibiting task-related activity during motor learning. Correlative EM reconstruction suggests that these functional clusters are hotspots for filopodia outgrowth to establish new connections with axons. The survival of the new spines depends on their co-activity with task-related spines in the vicinity, consistent with an earlier report that in the developing visual cortex, synapses “out of sync” with their neighbors are weakened over time (Winnubst et al., 2015). Theoretical studies have suggested that such clusters of synapses sharing similar activity patterns would enable the neuron to fully exploit the information processing capacity offered by dendritic nonlinearities (Kastellakis et al., 2023).

While the majority of *in vivo* imaging studies have focused on dendritic spines on M1 PyrNs, some researchers have endeavored to examine their inputs. Hasegawa et al. imaged the structural dynamics of axonal boutons in mouse M1 L1 during rotarod learning. They found an increased rate of bouton formation on axons from M2 from training day 2 to 4, and a decreased rate of bouton elimination on axons from the motor thalamus from training day 4 to 7 (Hasegawa et al., 2020). Combining *in vivo* 2P imaging with *post hoc* immunohistochemical validation, Sohn et al. found that motor learning-induced new spines receiving CC inputs tend to be transient, whereas those receiving TC inputs tend to enlarge and persist (Sohn et al., 2022). Together these works suggest that the transient CC connections facilitate motor learning, while the persistent TC connections store the established motor memory.

## 6. Contribution of inhibitory circuits to motor learning

The importance of inhibitory circuits in motor learning has been recognized for a long time. As astutely observed by Sir Charles S. Sherrington, “to refrain from an act is no less an act than to commit one, because inhibition is coequally with excitation a nervous activity” (Sherrington, 1933). In the mammalian brain, gamma-amino butyric acid (GABA) is the primary inhibitory neurotransmitter. To probe the role of inhibitory neurotransmission in motor learning, human studies have used Magnetic Resonance Spectroscopy (MRS) to measure local GABA level. One such study showed that anodal tDCS delivered to M1 induces a significant reduction in local GABA concentration, which is correlated with better performance in learning a force adaptation task (Kim et al., 2014). Other studies have probed inhibitory circuits by paired-pulse TMS (Kujirai et al., 1993; Ljubisavljevic, 2006). When a subthreshold conditioning stimulus delivered to M1 is followed by a suprathreshold test stimulus with a 1–6 ms inter-stimulus interval, the electromyography (EMG) response evoked by the test stimulus is inhibited. This phenomenon is known as short interval intracortical inhibition (SICI) and is considered a measure of GABA_A_ receptor-mediated inhibition (Ziemann et al., 1996a,b; Di Lazzaro et al., 2000, 2006, 2007). Several studies have reported a decrease in SICI associated with motor learning (Liepert et al., 1998; Perez et al., 2004; Smyth et al., 2010; Dupont-Hadwen et al., 2019), indicating a transient reduction in GABAergic inhibition putatively mediated through the GABA_A_ receptor. Taken together, these studies suggest that modulation of the inhibitory tone in M1 is crucial for human motor learning.

Rodent models enable researchers to dissect the contribution of inhibitory circuits to motor learning with cellular specificity. There are three major classes of inhibitory interneurons (INs) in the cerebral cortex: those expressing parvalbumin (PV+), somatostatin (SST+), and the serotonin receptor 5HT3aR. Among the 5HT3aR group, the most abundant are those expressing vasoactive intestinal peptide (VIP+) (Lim et al., 2018). Distinct subtypes of INs selectively synapse onto different domains of excitatory neurons as well as each other. For example, PV+ INs provide inhibition to the peri-somatic region of PyrNs, whereas SST+ INs inhibit distal dendrites of PyrNs (Fishell and Kepecs, 2020). A recent study combining rabies virus-based monosynaptic tracing and channelrhodopsin-assisted circuit mapping further shows that different types of M1 INs also exhibit distinct laminar profiles of inputs (Okoro et al., 2022). Using transgenic mouse lines that express Cre recombinase in specific subtypes of INs, Chen et al. found a subtype-specific plasticity of M1 inhibitory circuits during motor learning: axonal boutons of SST+ INs undergo elimination upon motor training initiation, while the number of axonal boutons of PV+ INs gradually increases through training (Chen et al., 2015).

The importance of SST+ INs in learning-associated synaptic reorganization is highlighted in other studies. While acute ablation or chemogenetic silencing of SST+ INs disrupts the branch-specificity of dendritic Ca^2+^ spikes and the associated synaptic potentiation on L5 PyrNs (Cichon and Gan, 2015), activating SST+ INs during motor training prevents learning-induced sequential activities of M1 L2/3 PyrNs and behavioral improvement (Adler et al., 2019). Notably, two recent studies suggest a transient reduction in SST+ IN activity level during the early phase of motor learning (Ren et al., 2022; Yang et al., 2022). This is reminiscent of an earlier study (Donato et al., 2013) that with environmental enrichment, a larger fraction of PV+ INs in the adult hippocampus show low PV expression; this state is related to enhanced structural synaptic plasticity, memory consolidation and retrieval. Together with human research, these studies suggest that a reduction in the inhibitory tone is prerequisite to the acquisition of a new motor skill.

## 7. Conclusion

Motor learning is a ubiquitous capability of mammals. While motor learning induces a plethora of concerted changes from the molecular to the systems level not only in M1 but also in other movement-related areas such as the anterior lateral motor cortex (ALM), M2, the striatum, and the cerebellum, structural and functional plasticity of the synaptic circuit is emerging as the leitmotif. At the same time, different species exhibit specific anatomical and physiological features dictated by their unique ethology and evolutionary history. Elucidating such variations on the common theme will greatly deepen our understanding of the neurobiological and computational basis of motor learning. To promote such cross-species studies, it is highly desirable to devise behavioral paradigms that are applicable to different species. Comparative studies on the transcriptomics, epigenetics, neuronal morphology, electrophysiology, and connectomics (Beaulieu-Laroche et al., 2018, 2021; Bakken et al., 2021; BRAIN Initiative Cell Census Network (BICCN), 2021; Loomba et al., 2022) will help distinguish conserved core features from species-specific innovations. Finally, there is a conspicuous gap in the accessible spatiotemporal scales in human vs. animal experiments. Many invasive techniques for probing and modifying the neural circuit at the molecular and cellular levels are not applicable to human studies for ethical reasons. Thus, it is important to develop novel non-invasive methods to study the human brain with synaptic and cellular resolution *in vivo*. Conversely, research on rodents and non-human primates very often focuses on one small subset of cells in one brain region at a time; optical and electrophysiological methods that retain the high resolution but cover larger volumes will be highly informative. Achieving these goals will require dialogues between neuroscientists working on humans and animal models, as well as interdisciplinary collaborations with experts in other branches of physical, biological, and applied sciences.

## Author contributions

EK: Conceptualization, Writing – original draft. JL: Conceptualization, Writing – original draft, Writing – review & editing. YZ: Conceptualization, Funding acquisition, Supervision, Writing – review & editing.
